# Toward Software-Equivalent Accuracy on Transformer-Based Deep Neural Networks With Analog Memory Devices

**DOI:** 10.3389/fncom.2021.675741

**Published:** 2021-07-05

**Authors:** Katie Spoon, Hsinyu Tsai, An Chen, Malte J. Rasch, Stefano Ambrogio, Charles Mackin, Andrea Fasoli, Alexander M. Friz, Pritish Narayanan, Milos Stanisavljevic, Geoffrey W. Burr

**Affiliations:** ^1^IBM Research–Almaden, San Jose, CA, United States; ^2^IBM T. J. Watson Research Center, Yorktown Heights, NY, United States; ^3^IBM Zurich Research Center, Zurich, Switzerland

**Keywords:** analog accelerators, BERT, PCM, RRAM, in-memory computing, DNN, Transformer

## Abstract

Recent advances in deep learning have been driven by ever-increasing model sizes, with networks growing to millions or even billions of parameters. Such enormous models call for fast and energy-efficient hardware accelerators. We study the potential of Analog AI accelerators based on Non-Volatile Memory, in particular Phase Change Memory (PCM), for software-equivalent accurate inference of natural language processing applications. We demonstrate a path to software-equivalent accuracy for the GLUE benchmark on BERT (Bidirectional Encoder Representations from Transformers), by combining noise-aware training to combat inherent PCM drift and noise sources, together with reduced-precision digital attention-block computation down to INT6.

## 1. Introduction

State-of-the-art Deep Neural Networks (DNNs) have now demonstrated unparalleled accuracy performance across a wide variety of fields, including image classification, speech recognition, machine translation, and text generation (LeCun et al., 2015). While current models are generally trained and run on general-purpose digital processors such as CPUs and GPUs, the rapid growth in both size and scope of these networks has fostered novel hardware architectures aiming to optimize speed and energy-efficiency, specifically targeting either neural network training or inference (Sze et al., 2017).

**Figure 1:**
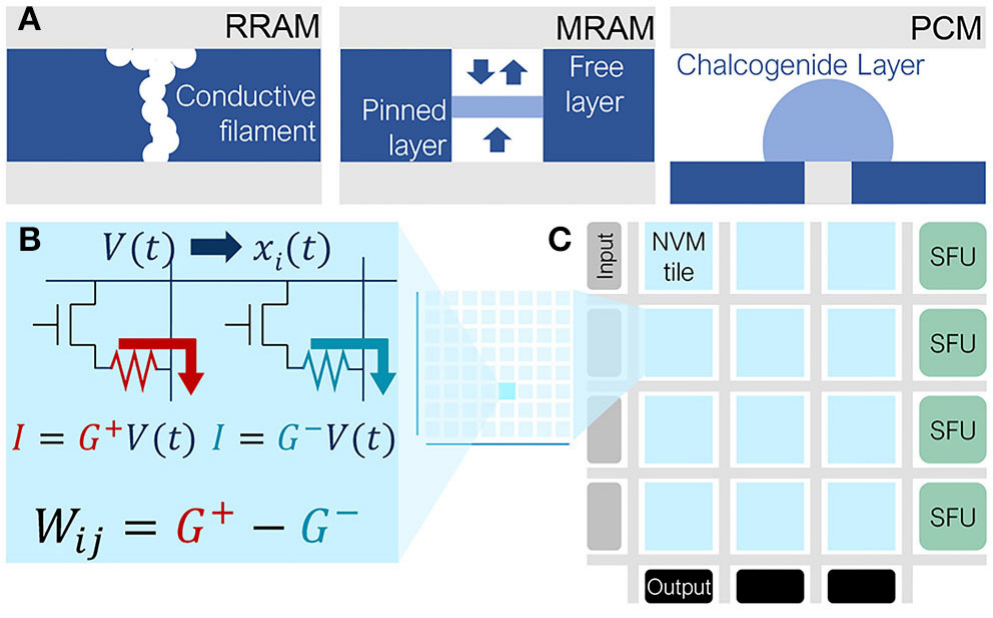
RRAM, MRAM or PCM devices **(A)** can be organized in crossbar arrays, or NVM tiles, where weights are encoded using pairs of devices **(B)**. Analog accelerators composed of multiple NVM tiles and special function units (SFU) for digital computation enable end-to-end network inference **(C)**.

Among these, architectures based on Non-Volatile Memory (NVM) are increasingly gaining interest. Such technologies encode weight information in the conductance states of two-terminal devices — including Resistive RAM (RRAM) (Wong et al., 2012), using modulation of conductive filaments between electrodes, or Magnetic RAM (MRAM) (Matsukura et al., 2015), using ferromagnetic switching between parallel or antiparallel spin polarization. In particular, Phase-Change Memory (PCM) (Burr et al., 2016) is based on thermally-driven reversible transitions between amorphous and crystalline states of a chalcogenide layer, leading to low and high conductances, respectively (Figure 1A).

Analog accelerators leverage the massive parallelism of NVM-based crossbar arrays to perform computation at the location of data (Burr et al., 2017; Ambrogio et al., 2018; Figure 1B). This architecture can significantly mitigate the Von-Neumann bottleneck caused by communication between the processor and memory, and is particularly efficient for fully-connected neural network layers (Burr et al., 2015).

A recent development in DNN-based natural language processing (NLP) is the migration away from recurrence toward Transformer-based models such as BERT (Bidirectional Encoder Representations from Transformers) (Devlin et al., 2018). BERT offers state-of-the-art performance over a wide range of Natural Language Processing (NLP) tasks. While the large fully-connected layers in these models are computationally expensive for both conventional hardware and custom digital accelerators, they are ideally suited for analog NVM-based hardware acceleration. However, NVM devices exhibit many conductance instabilities [conductance drift (Ambrogio et al., 2019), programming and read noise (Tsai et al., 2019), etc.], which can degrade accuracy, particularly as the time between programming and inference increases.

In this paper, after a brief overview of Transformer-based models including BERT, we use a device-aware simulation framework to develop and assess techniques that can increase the inference accuracy of BERT implemented using PCM devices. We show that these techniques allow these inherently fast and energy-efficient systems to also approach software-equivalent accuracy [as compared to the original BERT implementation (Devlin et al., 2018)], despite the significant noise and imperfections of current PCM devices. Since the high energy-efficiency of analog crossbar-arrays on the fully-connected layers will then expose the energy-inefficiency in digital computation of the attention blocks, we explore the impact of quantized attention-block computation. We show that the use of reduced precision down to INT6 can provide further energy optimization for Transformer-based models, applicable both to analog NVM-based as well as to other accelerator systems.

### 1.1. Transformer Architecture

The Transformer architecture (Vaswani et al., 2017) was a pivotal change-point in deep learning and is expected to remain a critical core as new models [BERT (Devlin et al., 2018), DistilBERT (Sanh et al., 2019), (Albert Lan et al., 2020), etc.] continue to build upon the underlying Transformer architecture. Here we describe how the Transformer architecture differs from recurrent DNNs, and how the basic building blocks of Transformers map to analog accelerators.

#### 1.1.1. Why Transformer?

**Figure 2:**
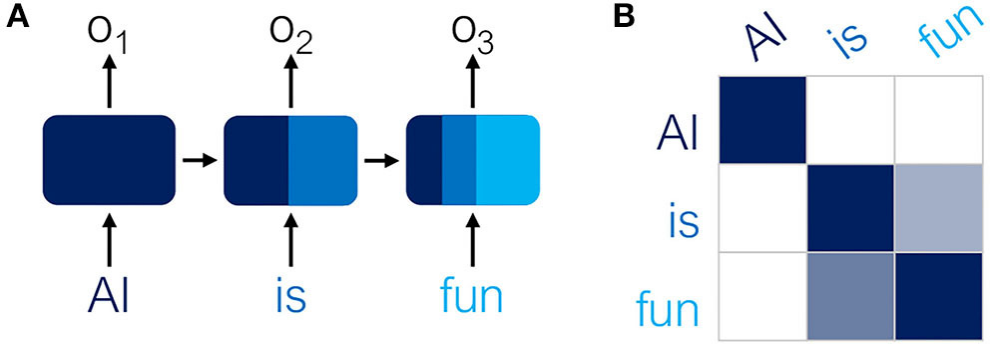
**(A)** Recurrent Neural Networks (RNNs) use recurrence to maintain “memory” of the sequence. Hidden states of previous words contribute to the next state. **(B)** In contrast, Transformers compute an attention matrix, where higher (darker) probabilities indicate which words are interrelated.

Recurrent neural networks (RNNs) have commonly been used for NLP tasks to account for the sequential nature of words and sentences (Figure 2A). The bottleneck of RNNs is their limited “memory” over very long sequences. Transformers (Vaswani et al., 2017) provide one solution by replacing recurrence with a self-attention mechanism. For any given word *w* in the sequence, an attention probability between 0 and 1 is computed between *w* and every other word in the sequence (Figure 2B), allowing the model to quantify the relative importance that each word has in predicting *w*.

#### 1.1.2. BERT-Base Model Architecture

**Figure 3:**
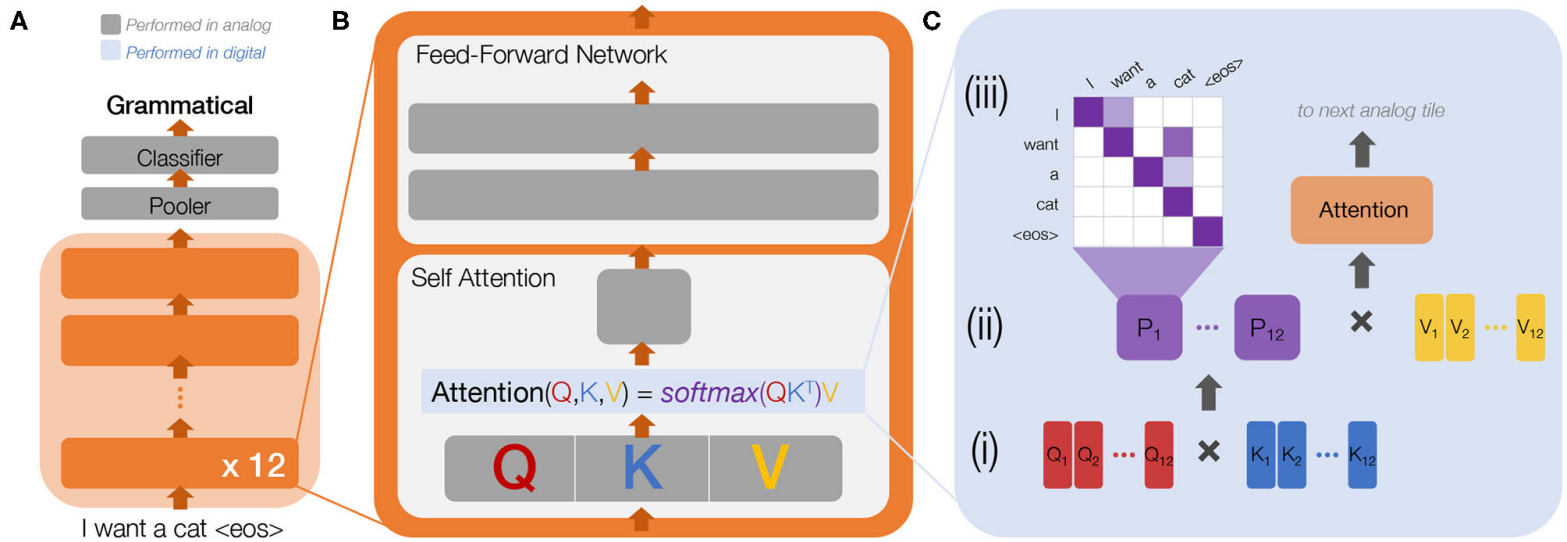
**(A)** Bidirectional Encoder Representations from Transformers (BERT) with 12 encoder layers. The input to BERT is a sequence of tokens, where each token is either a word or a word-piece. This sequence is processed through each layer, followed by a pooler to reduce output size and a fully-connected classifier layer. For example, to classify “I want a cat <eos>" (where <eos> is the end-of-sentence token) as either grammatical (0) or not (1), the classifier needs only two outputs. Each encoder layer **(B)** is comprised of two main building blocks: (1) the **self-attention block**, where the model computes an attention matrix between the input and itself, and (2) a **feed-forward network** with two large fully-connected layers. Dark grey represents trained weight layers in analog, while **(C)** shows the attention processing in digital. The input sequence to the self-attention block passes through a trained weight layer split into three parts to compute **Q** **(query)**, **K** **(key), and** **V** **(value) matrices**. To compute attention **(C)**, *Q*, *K*, and *V* are each split into multiple attention heads (for BERT, 12), both to reduce matrix sizes and to allow each to learn slightly different representations of the sequence. [c(i)] A similarity matrix is computed between *Q* and *K*, followed by a softmax operation along rows to produce values between 0 and 1. [c(ii)] These probabilities are then multiplied by *V* and move to the next analog tile followed by the feed-forward network. [c(iii)] A higher probability (darker shade) in one of the 12 probability (P) matrices might indicate, for example, that the word “cat” is important for prediction of the word “want”.

Building on the initial success of Transformers, BERT was developed to generate meaningful encodings of input sequences useful across a broad range of downstream tasks, such as classification, text generation, and machine translation, requiring only a few epochs of subsequent fine-tuning to prepare for the specific task. BERT consists of 12 layers of a large Transformer encoder (Figure 3A). In Figure 3B, detailing the main building blocks of each encoder layer, dark grey boxes represent trained weight-matrices (fully-connected layers) that can readily be mapped to analog crossbar arrays. The attention computations (Figure 3C) along with all activation functions (representing a small fraction of the total operations) are computed in digital processing units.

## 2. Materials and Methods

### 2.1. Optimizing Analog Accuracy for BERT

In this section, we first describe the comprehensive analog tile model used in this paper to capture realistic PCM crossbar array behavior. We then describe our simulation procedure and datasets for evaluation before discussing inference accuracy results. The simulator is implemented using a modified pytorch framework (Paszke et al., 2019) (including Caffe2).

#### 2.1.1. Analog Tile Model

Weights, in this study, are encoded using a differential conductance pair *G*^+^ and *G*^−^ without any redundancy scheme. Zero weights are encoded with *G*^+^ = *G*^−^ = 0, therefore considering both devices at the RESET (lowest) conductance of the analog device. While, in practice, the minimum conductance cannot be zero, therefore the accuracy of the zero conductance could be limited, the large (100x–1,000x) PCM device on-off ratio ensures a fairly good approximation of a zero weight with very low RESET conductance and RESET noise.

Multiplication in the analog tile is performed by tuning the input voltage pulse-width, to prevent distortions due to conductance non-linearities as a function of read voltage (Chang et al., 2019). In order to accurately simulate the analog components in the analog accelerator system, we include various sources of non-ideality in the analog multiply-accumulate (MAC) operation, including quantization errors within the digital peripheral circuitry and conductance noise within the analog NVM devices. In this section, we describe the PCM-based device noise model and optimized design parameters we used to achieve near software-equivalent accuracy inference on BERT.

#### 2.1.2. Programming Noise, Conductance Drift and 1/f Read Noise

**Figure 4:**
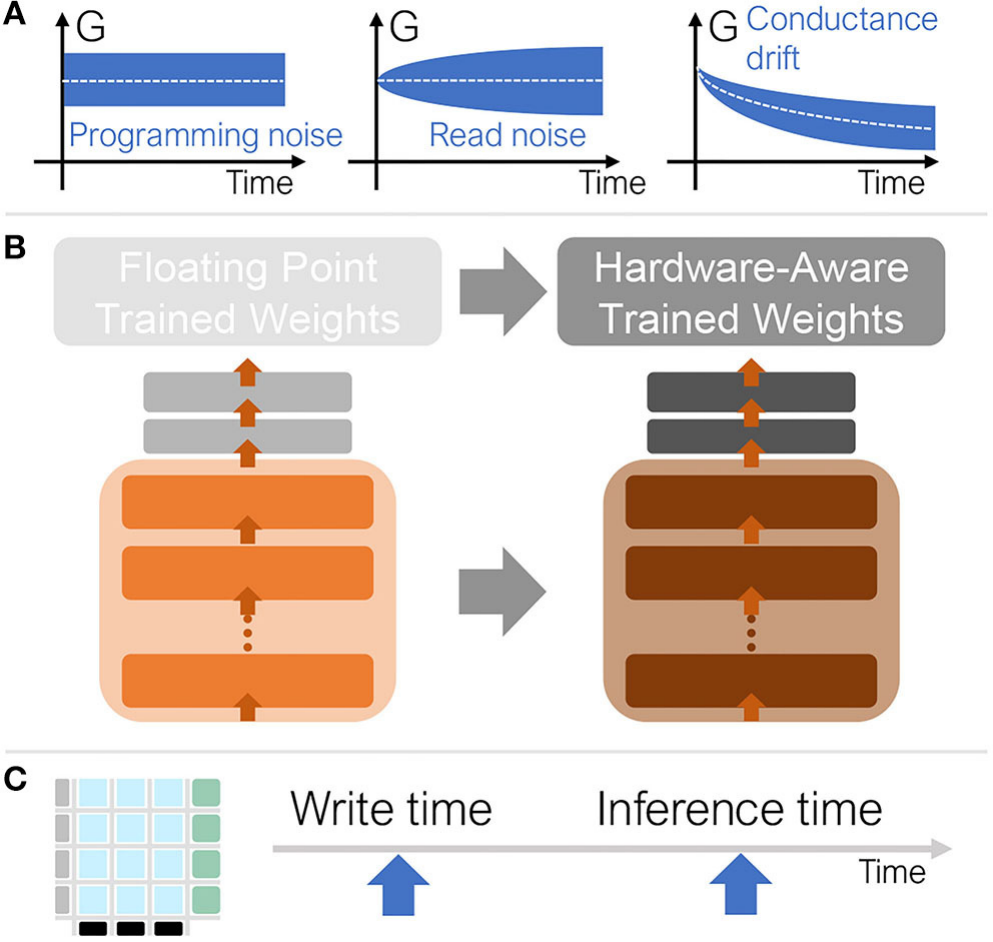
**(A)** Conductance values (G) exhibit variability due to programming and read noise and decay toward zero over time due to drift noise. **(B)** To mediate these noise sources, we train the floating-point model with noise in order to prepare the model for noisy inference. **(C)** During inference, the weights are programmed, then some time passes before inference is performed.

The inference accuracy attainable in an analog accelerator system depends strongly on the analog device conductance properties, since these can be noisy and change over time. In order to estimate the accuracy characteristics of future analog accelerators, we model these effects by adding programming noise, read noise, and conductance drift to the DNN weights (Figure 4A). We aggregate model error over many simulation instances to arrive at the expected inference accuracy for a given time point. The noise model used here is based on the experimental characterization from Joshi et al. (2020), with PCM devices fabricated in a 90 nm technology. The associated open-source simulator (Rasch et al., 2021) includes the following PCM statistical model for inference:

- Programming noise represents the error incurred when encoding the weight in the PCM device. Instead of programming the correct target, the final achieved conductance generally shows some error, which is modeled based on the standard deviation of the iteratively programmed conductance values measured from hardware (Joshi et al., 2020):
  $$\begin{array}{c} g_{prog}=g_{T}+N(0,\sigma_{prog}) \end{array}\;\left(\mu \text{S}\right)$$
  $$\begin{array}{c} \sigma_{prog}=\gamma max(1:1731g_{T}^{2}+1.965g_{T}+0.2635,0) \end{array}\;\left(\mu \text{S}\right)$$
  where *g*_*prog*_ and *g*_*T*_ are the programmed and target conductances of a PCM device and *N*(0, *σ*) is a normal distribution with standard deviation *σ*. The parameter *γ* is generally equal to 1, except when we explore the performances of devices with reduced noise, where *γ* = 0.5.
- PCM devices show a common trend for increasing time: after programming, due to the relaxation of the amorphous state, conductance decays, following an empirical power-law function expressed as in Ielmini et al. (2007):
  $$\begin{array}{c} g_{drift}(t)=g_{prog}{\left(\frac{t}{t_{c}}\right)}^{-\nu } \end{array}\;\left(\mu \text{S}\right)$$
  where *g*_*prog*_ is the programmed conductance measured at time *t*_*c*_ and *g*_*drift*_(*t*) is the conductance at time *t*, while *ν* represents the drift exponent, or slope on a log-G vs. log-t plot. In our simulations, *ν* is sampled from a normal distribution *N*(*μ*_*ν*_, *σ*_*ν*_). Both *μ*_*ν*_ and *σ*_*ν*_, dimensionless, depend on the target conductance *g*_*T*_ and are modeled by fitting experimental data from Joshi et al. (2020), with the following expressions:
  $$\mu_{\nu }=min(max(-0.0155log(g_{T})+0.0244,0.049),0.1)$$
  $$\sigma_{\nu }=min(max(-0.0125log(g_{T})-0.0059,0.008),0.045)$$
- PCM non-idealities also include instabilities after the programming stage, such as read noise. Even in the absence of programming error or conductance drift, consecutive PCM reads lead to slightly different conductance evaluations (Ambrogio et al., 2019). Among the multiple causes generating read noise, 1/*f* noise and random telegraph noise show the strongest contributions, with increased noise on lower-frequency components. Such behavior leads to analog levels' intrinsic precision degradation for longer times. The overall contribution can be modeled using a normal distribution with time-dependent sigma (Joshi et al., 2020):
  $$\begin{array}{c} g(t)=g_{drift}(t)+N(0,\sigma_{nG}(t)) \end{array}\;\left(\mu \text{S}\right)$$
  The standard deviation of the read noise σ_*nG*_ at time *t* is obtained by integrating the power spectral density over the measurement bandwidth:
  $$\begin{array}{c} \sigma_{nG}(t)=\gamma g_{drift}(t)Q_{s}\sqrt{log\left(\frac{t+t_{read}}{2t_{read}}\right)} \end{array}\;\left(\mu \text{S}\right)$$
  where *t*_*read*_ = 250 ns is the duration of the read pulse. The parameter *Q*_*s*_, dimensionless, measured from the PCM devices as a function of *g*_*T*_ is given by:
  $$Q_{s}=min\left(\frac{0.0088}{g_{T}^{0.65}},0.2\right)$$

The noise model used in this work was calibrated using a large number of PCM devices to characterize the statistics of (1) the weight programming error (due to deviations between programmed and desired conductance values), (2) the accumulated 1/f read noise of their PCM devices, and the (3) conductance drift and (4) drift variability as a function of the programmed conductance value. Details of the device measurement and modeling methodologies are described in the supplementary information of reference (Joshi et al., 2020).

#### 2.1.3. Analog MAC Design and Additional Non-Idealities

While weights are encoded using full precision, we include all noise sources, therefore reflecting the true analog nature of devices, we assume that each analog tile receives digital inputs at full precision, scales and quantizes to an integer representation, then converts to analog duration using digital to analog converters (DACs). The output of the analog tile is discretized using analog to digital converters (ADCs). Both DAC and ADC discretize the values in a fixed range symmetrically around zero. We assume 8 bit precision for DAC and 10 bit for ADC. The input scaling factor for the DAC is initialized using example data, learned during training to optimally match the input ranges, and kept static during inference. Target weight ranges are clipped to −1.0, …, 1.0, where 1.0 corresponds to maximum target device conductance, *g*_max_, although programming noise can induce overshoot. The output ADC range is related to the ADC gain and a parameter that depends on the ADC design. Here we set it to −10, …, 10, which means that 10 “fully on” input lines (each at 1.0) in conjunction with 10 weights at maximum (also 1.0) would saturate the ADC output. Even though the tiles have 512 rows, not all weights are at their maximum. In typical DNN models, most weights and activations have low values or are near zero. In addition, the random-walk nature of aggregation along the bitlines causes the signal to grow as the square-root of the number of rows, not linearly. The dynamic range of 10 for the ADC is a design parameter.

Each digital output from the ADC is individually scaled and offset, to map the conductances back to the high-precision digital domain (bfloat16 precision). These digital scaling factors are also learned during training and are critical to achieving software-equivalent accuracy during inference.

The analog MAC output is subject to short-term conductance-dependent noise that scales with the input current using the PCM read noise statistical model. We assume that the analog MAC output is subject to further additive Gaussian noise corresponding to 0.5 LSB (least significant bit) of the ADC, and use an approximated IR drop model. The analog tile size is set to 512×512 which, together with reduced read voltage (e.g., 0.2 V) ensures negligible IR drop impact; if layers are larger, they are distributed across multiple tiles and outputs are summed (in digital). Activation functions are computed in floating point 32-bit (FP32) format using standard functions.

### 2.2. Simulation Procedure–Training and Inference

Training for inference (i.e., hardware-aware training, or HWA) is done in software to make the subsequent hardware inference more robust, even in the presence of PCM non-idealities (Figure 4B). We apply noise during hardware-aware training, specifically during the forward propagation. While this helps the subsequent inference even in the presence of drift, this noise during training does not itself incorporate any explicit drift models. The subsequent backward propagation and weight update components or various scaling factors (described in previous sections) of software training are based on stochastic gradient descent (SGD) and are both carried out at full precision without additional noise.

Then, during inference, all hardware non-idealities—MAC cycle-to-cycle non-idealities, PCM programming noise, read noise, 1/f noise, drift, and drift variability—are considered, and drift compensation is applied as described below.

**Figure 5:**
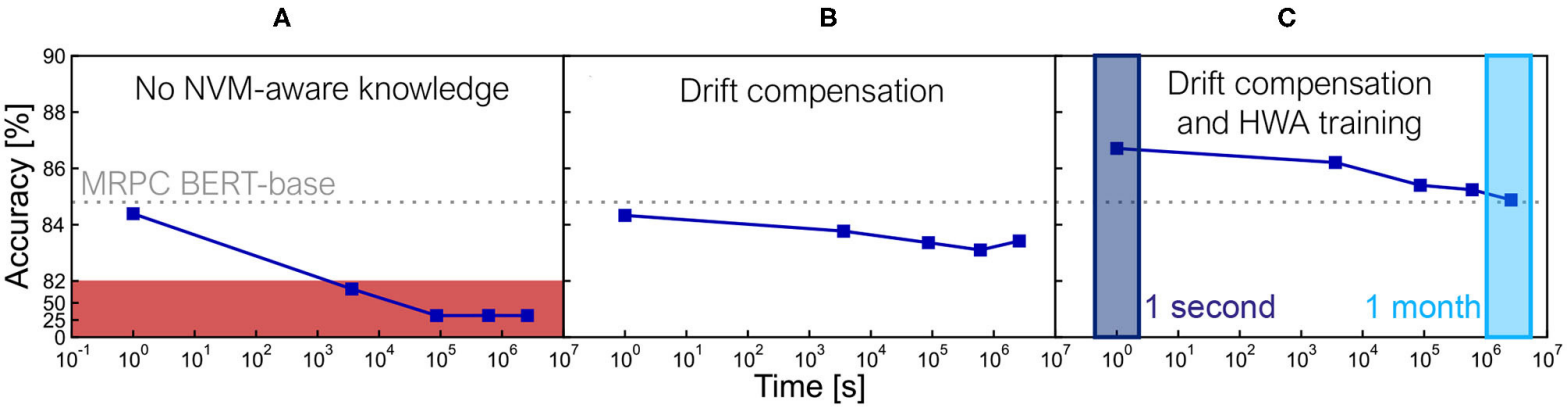
**(A)** Without any noise-aware techniques, inference on the Microsoft Research Paraphrase Corpus (MRPC) task decays very quickly over time. **(B)** Drift compensation improves the decay over time significantly, but the inference results are still lower than the BERT-base ideal model with no NVM noise. **(C)** Hardware-aware (HWA) training with noise added during training helps close the gap, reaching software-equivalent accuracy for this task even at 1 month of drift.

**Figure 6:**
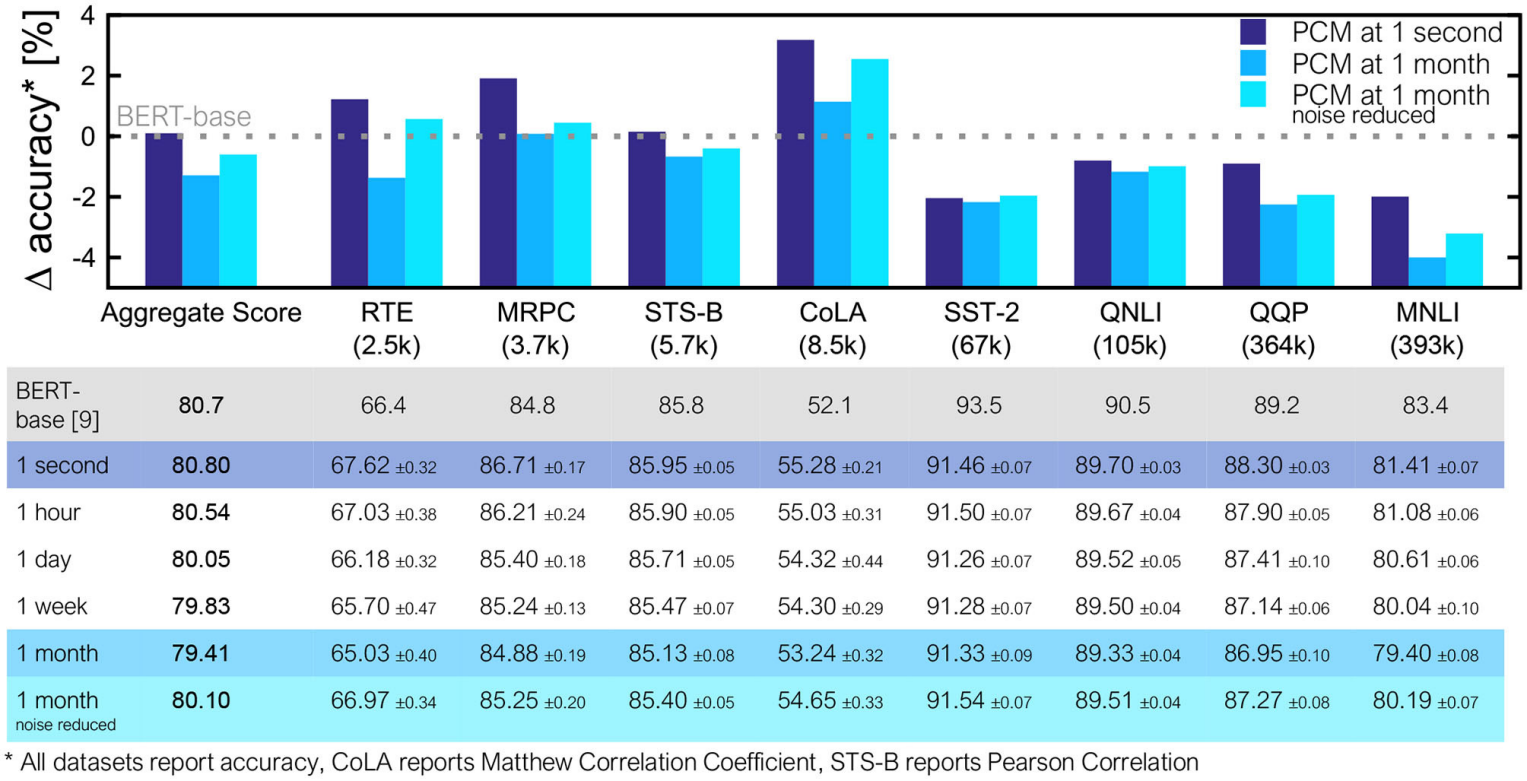
Inference results for all 8 GLUE tasks and the average score. Dataset training size shown in parentheses below each task name, and tasks appear in order of their size, with smallest on the left. Since each task has a different standard accuracy range, shown is the Δaccuracy between the results from the BERT-base model and our noise-aware trained model for two conditions: (i) full noise model applied, and (ii) 50% programming and read noise and full drift noise applied (noise reduced). For the full noise model, we consider several different time points, ranging from 1 month down to 1 day (with 1 hour and 1 second shown for context). The required time span would depend on the application. The table reports mean values across trials and standard errors of the mean.

We train 5 models with different random seeds and select the best one for inference evaluation. Accuracy can sometimes exceed state of the art results for smaller datasets where run-to-run variation can be wider, while larger datasets show smaller accuracy variation. We re-evaluate each model 25 times for each inference time point^1^ to reduce sampling error during inference. We also report the standard error in the tables of results (**Figures 6**, **8**). We evaluate accuracy at 5 time points after weight programming (Figure 4C): 1 second, 1 hour, 1 day, 1 week, and 1 month. Without any correction techniques, the inference accuracy drops markedly over time (Figure 5A).

#### 2.2.1. Drift Compensation

As described in Ambrogio et al. (2019) and Joshi et al. (2020) and illustrated in Figure 5B, signal loss by PCM conductance drift can be effectively compensated using a global correction-factor calculated from the mean drift over time. To calculate the drift compensation factor in the simulator, we first read out the weight matrix of each analog tile by performing the non-ideal MAC operations of the forward pass using one-hot input vectors, summing the values in an absolute manner to obtain an initial reference value. Then after applying conductance drift and accumulated 1/f noise to the weights up to a certain inference time-point, the weights are again read out through the same (non-ideal) MAC operations to produce a delayed reference value. Drift compensation is applied by adjusting the digital output scale-factor (applied after ADC) by the ratio of the delayed and initial reference values, and applied across the entire test set for all simulations of the model at that inference time-point. Once the average drift is compensated, the remaining noise effects act as a random walk process, as programmed conductances evolve away from their intended states. RRAM, FERAM, or any other device will also exhibit time-dependent conductance change, and these devices can also benefit from the methodology proposed in this work by substituting the corresponding device noise models.

#### 2.2.2. Hardware-Aware (HWA) Training

Drift compensation helps with the accuracy decrease over time by boosting the signal, but cannot remove the underlying noise sources. In addition to training the static scale factors for DAC input and ADC output, we apply a variety of techniques to prepare our trained model for noise during inference (Gokmen et al., 2019; Joshi et al., 2020). A noise model that includes digital periphery noise and additional noise on DNN weights that mimics a scaled version of our programming noise is applied during training, to prepare the network for inference with noisy weights. The standard deviation scale of this additional weight noise is a hyper-parameter of the HWA training. The effects can be seen in Figure 5C, reaching software-equivalent accuracy for a single language task only once these HWA training techniques are applied.

### 2.3. Datasets and Training

We evaluate our HWA-trained BERT on the General Language Understanding Evaluation (GLUE) Benchmark (Wang et al., 2019), consisting of 9 primary language tasks (see leaderboard at Wang et al., 2020). This benchmark is more robust than examining a single task, as it shows the network's ability to generalize. For example, one task tests the network's ability to identify a given sentence as grammatical or not. Another task assesses, given two sentences A and B, whether A is a paraphrase of B. We exclude one task, Winograd Natural Language Inference (WNLI), just as BERT (Devlin et al., 2018) did, due to the unusual construction of the data set and small test set of only 146 samples. This leaves 8 tasks:

- Microsoft Research Paraphrase Corpus (**MRPC**) (Dolan and Brockett, 2005)
- Recognizing Textual Entailment (**RTE**) (Bar-Haim et al., 2006; Dagan et al., 2006; Giampiccolo et al., 2007; Bentivogli et al., 2009)
- Semantic Textual Similarity Benchmark (**STS-B**) (Agirre et al., 2007)
- The Corpus of Linguistic Acceptability (**CoLA**) (Warstadt et al., 2018)
- The Stanford Sentiment Treebank (**SST-2**) (Socher et al., 2013)
- Question Natural Language Inference (**QNLI**) (Rajpurkar et al., 2016)
- Quora Question Pairs (**QQP**)
- Multi-Genre Natural Language Inference (**MNLI**) (Williams et al., 2018)

We evaluate each task separately by fine-tuning a pretrained BERT-base model (Wolf et al., 2020) using our HWA training techniques. We do not train BERT models from scratch using HWA training, but instead perform fine-tuning from the pretrained BERT model checkpoint with these techniques. Fine-tuning is a technique used in natural language processing, similar to transfer learning, where the main model is trained with a large amount of generic language data and later fine-tuned for a specific task (e.g., sentiment classification) using a much smaller set of data with limited epochs of training. This greatly reduces the runtime for the HWA training when compared to training from scratch. We use a maximum sequence length of 128 for efficiency, since the vast majority of data samples are much shorter that the maximum BERT sequence length of 512. We report the aggregated score of all 8 tasks, since this is a common metric reported for GLUE (Wang et al., 2019).

Each task needs to be fine-tuned differently, so we scanned a variety of learning parameters for each task: batch size, learning rate, weight clipping, and dropout. Here we report the accuracy on the validation data set because the test set is only available online, which might result in a slight overestimation in the accuracy scores for the datasets with small validation set. We observe accuracy variation that correlates with the size of the datasets—models trained with smaller datasets exhibit larger variation in test accuracy. Therefore, we train 5 models per task per condition and choose the best model for inference simulation.

## 3. Results

### 3.1. Results on BERT

Figure 5C shows an example of an HWA-trained BERT-base model reaching software-equivalent accuracy and the inference accuracy evolution over time for the MRPC task. Accuracy results on all 8 GLUE tasks, reported at times ranging from 1 second to 1 month after weight programming, are summarized in Figure 6. We show that several tasks reach software-equivalent accuracy at 1 month and the biggest accuracy drop is ~4% for MNLI. The aggregate score over all 8 tasks is only 1.29% below the baseline at 1 month. Since there is hope for additional improvement with progress in PCM device technology (Giannopoulos et al., 2018), we show results for the full drift model but with only 50% of the programming and read noise applied during inference, achieved by setting the γ factor in the σ_*prog*_ and σ_*nG*_(*t*) equal to 0.5. In this way, we reduce the impacts of both programming and read noise contributions. Noise-reduced PCM devices can be expected to improve many of the tasks by >1% even for inference after 1 month, and increase the aggregate GLUE score to just 0.6% below baseline.

### 3.2. Attention Quantization

**Figure 7:**
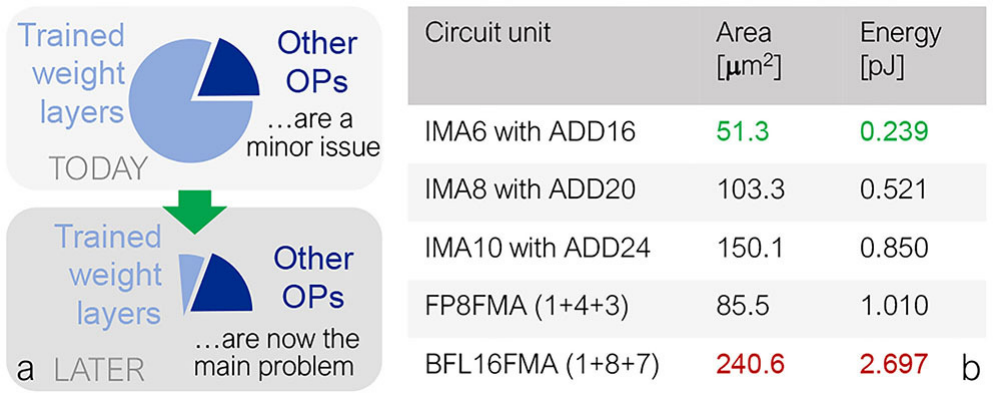
**(A)** While the energy-inefficiency in high-precision digital computation of the attention blocks may currently be a minor issue, the high energy-efficiency of analog crossbar-arrays on fully-connected layers will eventually expose this as a problem. **(B)** Particularly in Transformer-based models, quantizing the attention block to lower precision greatly reduces the area and energy usage of the multipliers, optimizing the new bottleneck: activation processing in attention. For example, decreasing from bfloat16 (“BFL16FMA”) to INT6 (“IMA6”) results in an estimated energy reduction of 91%.

Attention-based models such as BERT pose unique challenges beyond previously studied models, because of the extensive activation computation in the self-attention block. Amdahl's law implies that when a system bottleneck is greatly improved, performance is invariably limited by something else, no matter how insignificant it was to begin with (Figure 7A). Self-attention computations in a Transformer model scale quadratically with sequence length *S*, and constitute <1% of the number of operations for small *S*, but ~5% at *S* = 128. If this computation is done in digital processing units at full precision, the cost in both energy and area for such processing units can become the system bottleneck for Transformers, particularly as sequence length grows, despite constituting a relatively low fraction of the workload.

Reduction of the precision in the digital computation of this self-attention block can also help reduce overall computation costs, beyond consideration of the analog performance and precision of just the fully-connected layers. The attention matrix in this case is not mapped into analog crossbar arrays, but processed in digital multiply-and-add units.

#### 3.2.1. Attention Computation

In the self-attention block, there are two batch matrix-multiplies, one for Q*K and one for *softmax*(Q*K)*V (Figure 3C(i,ii)). In this paper, we propose to compute batch matrix-multiplication with various integer precisions in order to reduce energy and area costs for these attention computation units, while keeping softmax operations at full precision. When compared to bfloat16 multiply-and-add (BFLFMA), integer multiply-and-add (IMA) units are much more energy and area efficient. Figure 7B, simulated in a 14 nm FinFET technology, shows a 11.3× energy benefit and a 4.7× area benefit from BFLFMA to INT6 (including a wide-enough adder for multiply-accumulate operations across the 64 terms in an attention-head). Next, we explore the impact of these attention quantization options on inference accuracy in BERT.

### 3.3. Results on BERT With Quantized Attention

**Figure 8:**
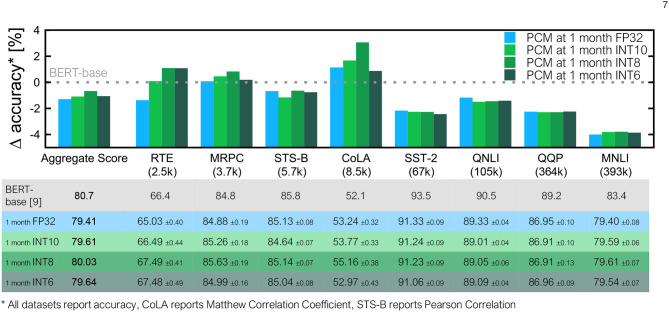
Quantization inference results for all 8 GLUE tasks and the average score. Shown is a comparison to our FP32 noise-aware model from Figure 6 at 1 month of drift for various levels of precision: INT10, INT8, and INT6, all of which perform at least as well as our full precision model for most tasks. On some tasks, including the aggregate score, the reduced precision seems to serve as additional regularization and performs better than our FP32 model. The table reports mean values across trials and standard errors of the mean.

Figure 8 summarizes GLUE task inference results with our analog tile models for the fully-connected layers with four different precision settings—FP32, integer 10 bit (INT10), integer 8 bit (INT8), and integer 6 bit (INT6)—for the batch matrix-multiplications in self-attention. The scaling factor used for quantization is initialized from a small set of training data and then learned during the training process. BERT inference performance is comparable among all four quantization schemes. For smaller datasets, INT10, INT8 and INT6 quantized attention models sometimes outperform the FP32 versions because of the additional regularization and noise in the attention layers during training. For the four larger datasets (SST-2, QNLI, QQP, and MNLI), no significant differences in inference accuracy at 1 month were observed down to INT6 quantized attention.

## 4. Discussion

While we have clearly demonstrated the potential for iso-accuracy with Transformer-based neural networks on fast and energy-efficient analog hardware, there are numerous areas for future work.

### 4.1. Software-Equivalent accuracy

We have shown that full software-equivalent accuracy will require continued improvement in both PCM devices and in hardware-aware training techniques. However, we have been reasonably conservative in our accuracy report, presenting results at 1 month of inference. We note that some workloads may only require results at 1 day or 1 week of drift, for example when models are weekly updated. We project that current PCM devices can comfortably support software-equivalent accuracy on many GLUE tasks on such timescales. For tasks where models are less frequently updated, another approach would be to incur slightly more frequent in-place reprogramming of the same model – this would be a tradeoff between model availability, the time needed for model programming, device endurance, temperature variation and other factors.

### 4.2. Model Size

While we have focused on BERT, which has 110 M parameters, new Transformer-based networks are emerging that attempt to reduce model size while maintaining accuracy. DistilBERT (Sanh et al., 2019) uses knowledge distillation to reduce the number of parameters in half, and ALBERT (Lan et al., 2020) uses cross-layer parameter reuse, reducing the number of unique parameters to a fraction of the original. However, we note that these smaller models may present a challenge to analog hardware, since fewer unique weights can make models less robust to noise. Hardware-software co-optimization that can strike a good balance between model size and robustness to PCM-based noise could lead to future Transformer-based networks that are highly optimized for accuracy, energy-efficiency, and speed on Analog-AI hardware.

## 5. Conclusion

We show that despite their various noise sources, PCM-based analog accelerators are a sensible choice for deep learning workloads, even for large natural language processing models like BERT. Our simulation results using a comprehensive noise model demonstrate that BERT can be expected to be close to software-equivalent accuracy even with existing PCM devices. Other Transformer-based models with the same building blocks can be similarly evaluated with our approach. We have shown that expected improvements in programming noise variability provide a consistent trend toward software-equivalent accuracy. Finally, in preparation for high energy efficiency on the fully-connected layers, we provide a potential solution to the next biggest energy cost: the activation processing from the attention block. We show that 11.3× energy improvements should be feasible by quantization to INT6, with no significant loss in accuracy.

## Data Availability Statement

The original contributions presented in the study are included in the article/supplementary material, further inquiries can be directed to the corresponding author/s.

## Author Contributions

KS, HT, MS, and GB conceived the original ideas. KS, HT, AC, and MS implemented and ran the simulations. All authors contributed during data analysis. KS, HT, AC, MR, SA, and GB drafted the manuscript.

## Conflict of Interest

The authors were employed by IBM Research.

## Footnotes

^1^For one particular task, Quora Question Pairs (QQP), we use only 5 repeats due to large test dataset size.
